# Advancing Education in Endoscopic Spinal Navigation: Novel Methods and Technical Note

**DOI:** 10.7759/cureus.37017

**Published:** 2023-04-01

**Authors:** Maria Eduarda Pertile, Yan de Assunção Bicca, Paula M Maccari, Orlando R Neto, Douglas P Quintas, Raphael Bertani, Sávio Batista, Stefan W Koester, Eloy Rusafa, Marcus Vinicius Flores de Barros Vasconcelos Fernandes Serra

**Affiliations:** 1 Medicine, University of Caxias do Sul, Caxias do Sul, BRA; 2 Spine Surgery, Instituto Santista de Neurocirurgia e Coluna, Santos, BRA; 3 Spine Surgery, Brazilian Endoscopic Spine Surgery Center (BESSC), Santos, BRA; 4 Neurosurgery, University of São Paulo, São Paulo, BRA; 5 Medicine, Federal University of Rio de Janeiro, Rio de Janeiro, BRA; 6 Medicine, Vanderbilt University School of Medicine, Nashville, USA

**Keywords:** minimally-invasive spine surgery, surgery spine, lumbar spine surgery, spine technology, innovative teaching learning, endoscopic spinal surgery, spine, endoscopic surgery, endoscopic navigation

## Abstract

This report aims to demonstrate how to teach anatomy and understanding of spinal endoscopic vision and navigation using mnemonics. The authors present a new surgical technique for teaching endoscopic spinal navigation in a didactic manner with tips such as the “rule of the hand” and decomposition of the endoscopic navigation movement. We demonstrate how the surgery is seen and illustrate how images are projected onto the screen, then divide the navigation into spatial orientation and self-navigation. The article describes the proper puncture technique, how to introduce the working portal, and how to assimilate this new anatomical vision using the “rule of the hand.” The surgeon projects their hand on the video screen to guide themselves when starting the navigation and uses the same technique to localize regions of interest during surgery. Finally, the authors break down the navigational movement into three components: forceps positioning, triangulation, and joystick motion.

One of the biggest challenges when learning spinal endoscopic surgery is understanding the anatomy seen through the endoscope. By decomposing movements required for navigation, one can understand how to make proper use of the equipment as well as improve their knowledge of this “new anatomy.”

The learning methods taught in this article have the potential to decrease the learning curve and radiation exposure to those that are still acquainting themselves to spinal endoscopic navigation. We recommend that further studies measure and quantify the impact of these methods on surgical practice.

## Introduction

Minimally invasive (MI) techniques are gaining popularity recently due to patient satisfaction and procedural feasibility with ease and effectiveness. Endoscopic navigation, an MI technique recently developed in spine surgery, was proven effective in the treatment of a range of pathologies, from degenerative diseases (such as lumbar disc herniation and foraminal stenosis) [1-4] to the treatment and biopsy of infectious and tumoral processes [5-7]. The technical training of the surgeon in this MI technique, however, has a steep learning curve [8-11] as well as a high financial burden involving investment in courses, instruments, and surgical mentorship [12].

Endoscopic spinal surgery (ESS) has provided a new way to understand and visualize the anatomy of the spine. ESS decreases the surgeon’s field of view but improves the surgeon’s visualization by bringing anatomic perspective into the patient’s body and moving visualization and light source closer to the surgical site [13]. During the learning phase of ESS, the less experienced surgeon will likely require more intraoperative imaging and, consequently, greater exposure to radiation for safer and more effective navigation for both the surgeon and the patient [14]. With that in mind, we have developed a practical and didactic teaching method based on anatomy for ESS.

## Technical report

Instrumentation and visualization

Before starting the surgical procedure, one must adequately know the instruments they are using and how the vision of such tools projects onto the screen. There are multiple configurations of an endoscope. However, common amongst all of them is their instrument’s distal four-part composition: the working channel, the serum inlet, the serum outlet, and the lens. The technique described below comes from adequately understanding the lens and its principles. The lens is responsible for the passage of light that will illuminate the anatomical structures. The reflection of this light will be captured by the mirror system, which will then be transmitted to the video system via an optical fiber, generating the images.

**Figure 1:**
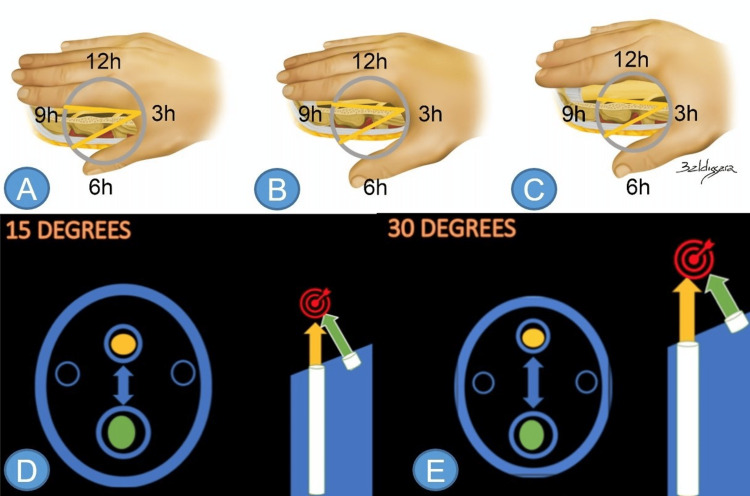
Angles of the transforaminal approach, and future point Different angles of an endoscope: 15 degrees (A), 25 degrees (B), and 30 degrees (C). Assuming that the endoscope is inserted 10 cm from the midline, the 15-degree endoscope allows sight of the emerging root (thumb) but not the midline. The 25-degree endoscope allows good visualization of the emerging root and the midline (the index finger in B represents the transversing nerve root and posterior longitudinal ligament, not just the transversing nerve root, as seen in A). The 30-degree endoscope allows excellent visualization of the transversing nerve root (index finger). Future Point projection on 15 (D) and 30-degree (E) endoscopes, respectively. Note that a more angled endoscope means a greater distance from the camera to the working channel, making the future point visible in surgery. Green arrow – working channel; Yellow arrow – light source; Target sign – future point projection. Source: author

On the distal portion of the endoscope, there are faceted lenses. The metal cut in this region defines the angle of the optics, which can be 15, 25, or 30 degrees. This allows visualization of the same point from different angles (Figures 1A-1C).

The spot where there is a crossing of the light and the instruments utilized in the procedure is called the future point. The distance between the working channel and the lens defines this point. Thus, constructing a zero-degree endoscope would make the procedure impossible due to the impossibility of projecting a future point to work with. In 15-degree endoscopes, the distance must be shorter, and in those of 30 degrees, longer (Figures 1D, 1E). This physical concept of relative distances and angles of instrumentation facilitates comprehension of the relationship between the entry point (distance from the midline to the surgical access site) and the optics.

**Figure 2:**
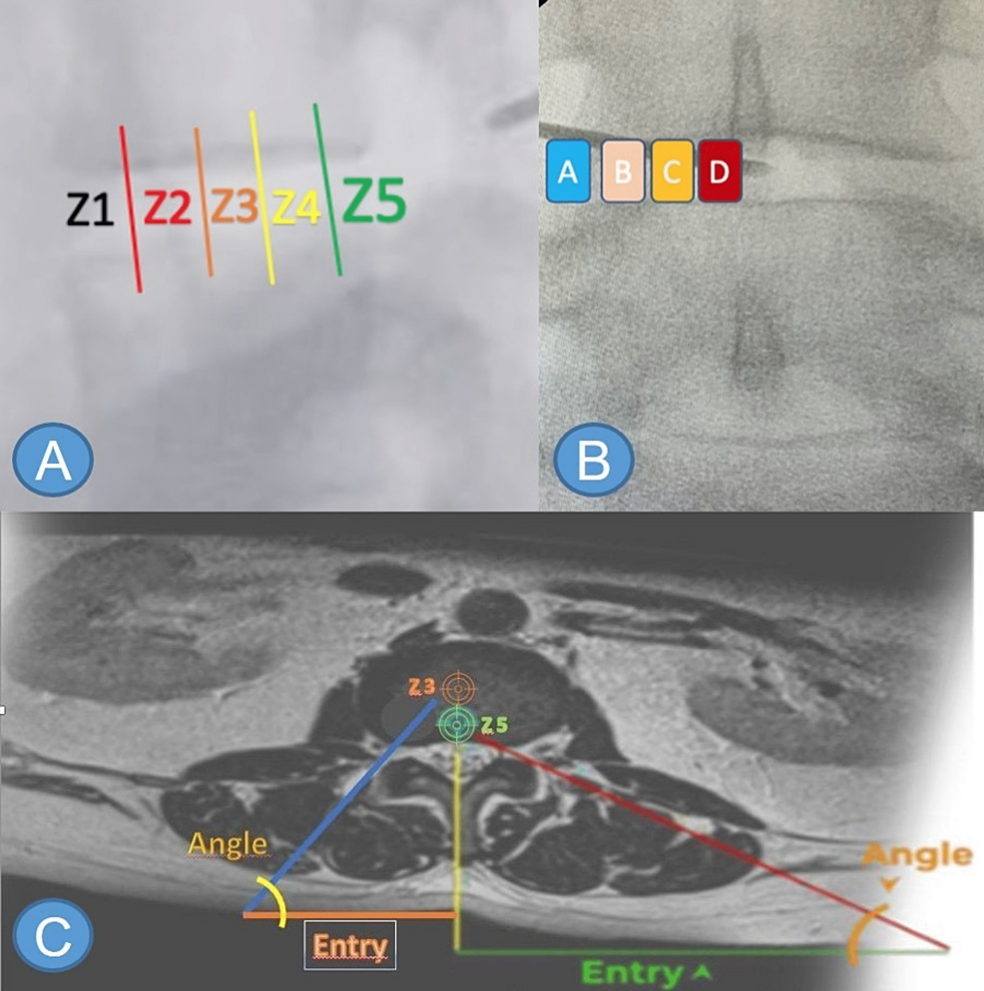
Intervertebral disc zoning, and entry point (A) lateral view radiography showing the lumbar spine. (B) Anteroposterior view radiography showing the lumbar spine. Z1 - "danger zone," close proximity to the abdominal cavity; Z2 - mid anterior; Z3 - center of the disc; Z4 - mid posterior; Z5 - adjacent to the canal; A - extraforaminal; B - foraminal; C - posterolateral; D – central. Zone 1 is adjacent to the abdominal structures and great vessels, Zone 3 is the center of the disc, and Zone 5 is the region close to the canal. (C) projection of the hernia's location (seen on MRI during intraoperative radioscopy). Puncture planning in the transforaminal access (T2 axial cross-section in MRI): Z3: center of the disc - foraminal and extra-foraminal hernias; Z5: adjacent to the canal - central and posterolateral hernias. Source: author

Another essential concept is the three-dimensional anatomical plane. The safety triangle [15] must be understood as it is not on the sagittal nor coronal plane but in the oblique plane. For better understanding, the authors divided navigation into spatial orientation and navigation. During the surgical procedure, in the sagittal plane, the vertebra is seen as if divided into five zones: Zone 1 (Z1) is adjacent to the abdomen, and Zone 5 (Z5) is adjacent to the canal (Figures 2A, 2B) [15]. Therefore, regarding herniated discs, the procedure must be performed as close as possible to Z5, as Z1 is a “dangerous” zone due to its proximity to the structures of the abdominal cavity.

Preoperative magnetic resonance imaging (MRI) is essential for initial spatial orientation. The calculations of the entry point and angle of puncture are made, taking noble structures along the pathway into consideration. In central and posterolateral hernias, the target is Z5, while in foraminal and extraforaminal hernias, the target becomes Z3. It is important to notice that this concept is only taken into account for puncture site estimation, as during the surgical procedure the workspace should be restricted to Z5 (Figure 2C).

The more lateral the puncturing needle enters the skin, the more medial it will enter the working space and vice versa. Therefore, when entering the skin medially, the facet throws the needle toward the lateral facet line without being considered an inadequate entry. Medial punctures keep the passerby root protected and expose the emerging root. In contrast, lateral punctures create distance from the emerging root and expose the passerby root since there is no protecting superior articular process from this angle.

**Figure 3:**
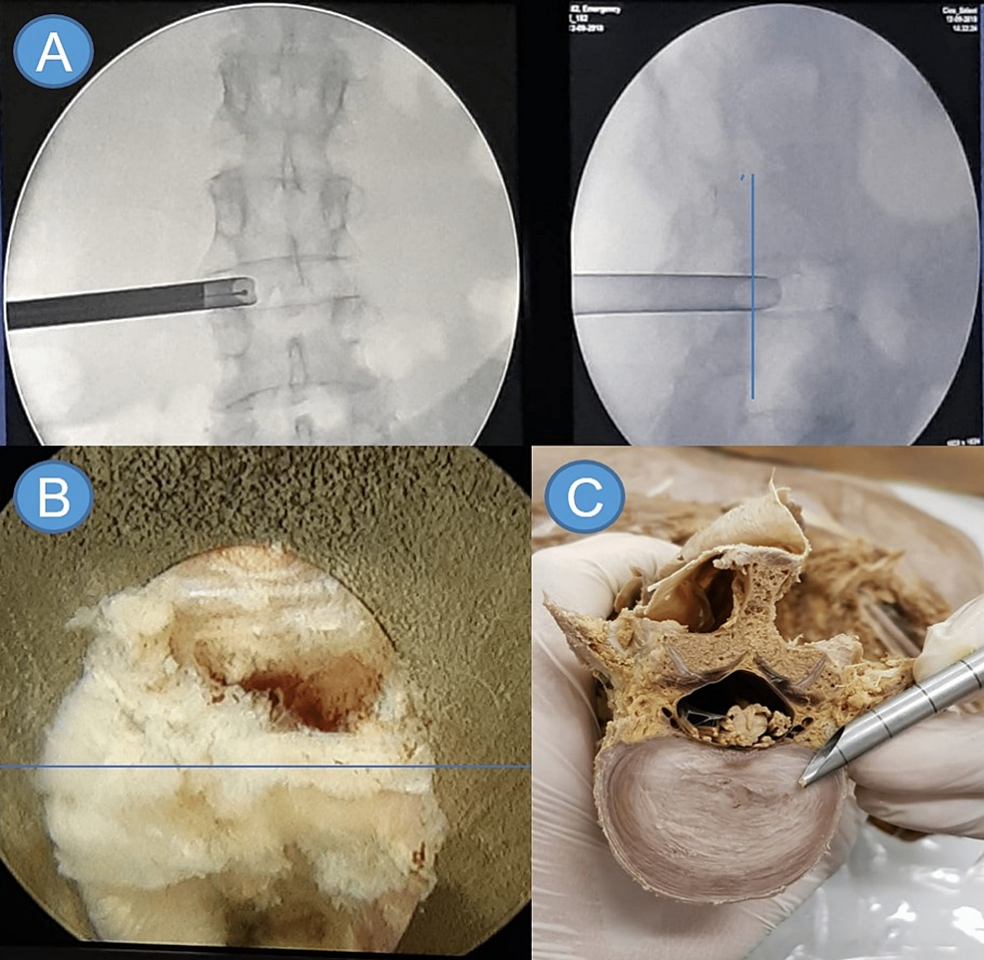
(A) Intraoperative imaging of the endoscope’s position (from left to right: anteroposterior and profile lumbar spine radiography); (B) endoscopic view of “half and half”; (C) representation of the endoscope’s position using an anatomical model (L2). Source: author

After the puncture, a few techniques are described to introduce the working portal. The working portal, when beveled, has a base and a point. One must understand the relationship between the working sheath and the anatomic structures seen on the video screen. Suppose the tip and the base of the bevel are anterior to the posterior line of the vertebral body in the lateral fluoroscopy when entering with the endoscope. In that case, the surgeon will have entered the disc. Retracting a few millimeters and performing a new fluoroscopy, the image of “half and half,” that is, the instruments advancing into “half” the disc, will be seen. Upon inserting the endoscope, we can observe a disc at 6 o'clock and the vertebral canal at 12 o’clock (Figures 3A-3C).

**Figure 4:**
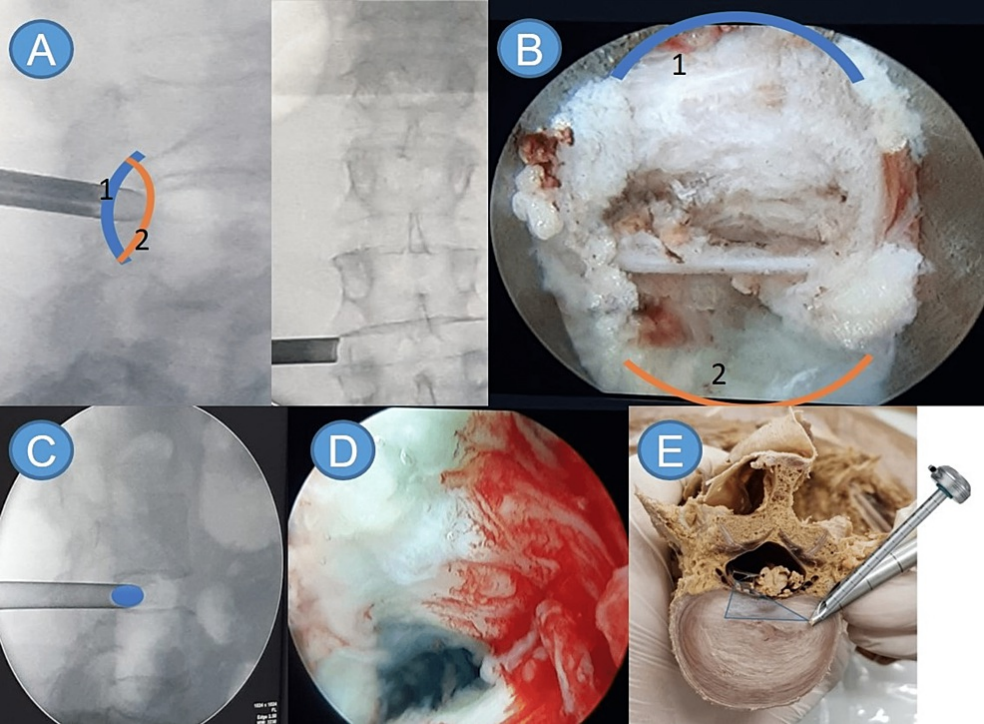
Radiological anatomy of the working sheath and endoscope facing the midline Configuration of the endoscopic view after retreating the endoscope a few millimeters from ‘’half and half". (A) Intraoperative imaging by profile radiography of the lumbosacral spine; (B) endoscopic view. A: bevel base; B: bevel tip. On the lateral radiography, the bevel base (A) is on the facet line, so when we look at the video screen, everything adjacent to it will be the facet. The bevel tip (B) is touching the disc, so on the video screen, the disc will be seen adjacent to it. (C) Intraoperative imaging confirming the endoscope’s position (profile lumbar spine radiography); (D) endoscopic view of the working area; (E) anatomical model representation (L2) of the correct position of the endoscope and the reference triangle that will appear on the video feed. Inside-out approach: the base and the tip are inside the disc and when entered with the endoscope, only the disc is observed. Source: author

If the endoscope retreats a few millimeters further, the tip of the bevel is positioned on the posterior line of the vertebral body, and the base on the articular facet. Thus, the endoscope will have the vertebral canal at 6 o’clock and the articular facet at 12 o’clock (Figures 4A, 4B).

In the “inside out” technique, the working portal (bevel tip) starts completely inside the intervertebral disc. During the procedure, the positioning of the surgical instruments can be verified through fluoroscopy, albeit at the cost of increased radiation exposure. In the “outside in” technique, the bevel tip will be on the anterior facet line and the base on the posterior line to see the facet and its joint with the pedicle. Both use the ‘’rule of the hand’’ and the interlaminar and stenosis approaches, each with its own rules. To assimilate this “new” anatomical vision, the authors propose the “rule of the hand.” The endoscope must face the patient’s midline, which means that the endoscope’s mark must also face the midline. The camera has to be aligned with the endoscope so that the reference triangle will be at 6 o’clock in the video feed (Figures 4C-4E). With this positioning of the endoscope and the camera, the anatomy of the video is the same that of the patient, facilitating navigation.

**Figure 5:**
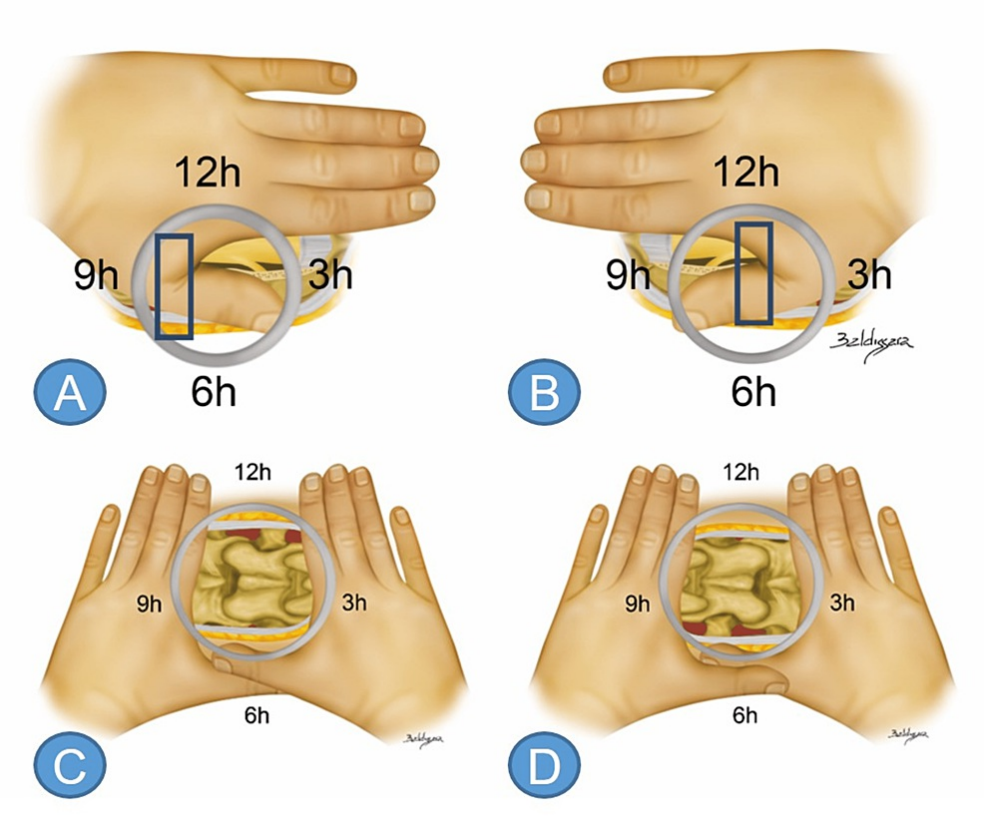
Interlaminar right and left-hand rule Anatomical model representation of the endoscopic view with each hand demonstrating orientation of the interlaminar window content. The thumb represents the transversing nerve root (in the L5-S1 space it is the root of S1; in the L4-5 space it is the root of L5) and the other fingers represent the dural sac (A). When on the patient’s left side (B), overlap the left hand over the right hand. The left index finger is the L4 lamina, the left thumb is the L4 inferior articular process; the right thumb is the L5 superior articular process, and the right index finger is the L5 lamina (C). When on the patient’s right side, swap the positioning of the hands (D). Source: author

In order to create an illustrative image for the surgeon, the “rule of the hand” was developed for many situations, including transforaminal, interlaminar, interlaminar bone window, and cervical approaches (Figures 5A-5D).

**Figure 6:**
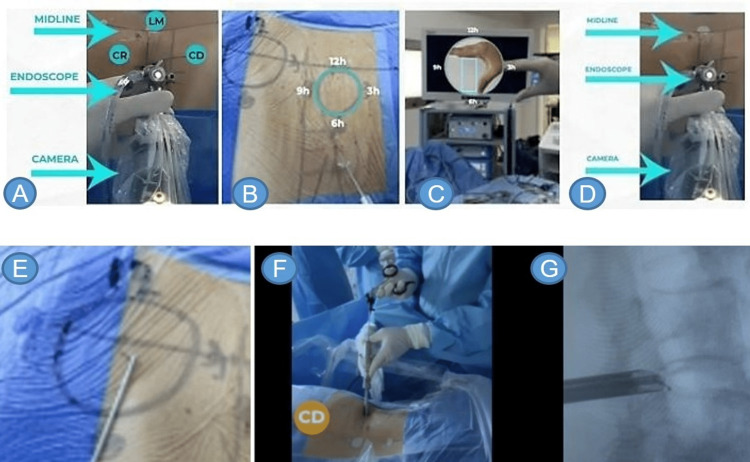
Clock position orientation Two clocks are depicted, one is drawn on the patient’s back (A, B), while the other clock is represented on the video screen (C). When the camera’s head (where the buttons are located) is faceted along with the anterior part of the endoscope (mark) and both are perpendicular to the midline, the anatomical references of these two clocks overlap (D). Representation of the anatomy of the patient (E-G); it is used for spatial orientation, both by allowing redirection of the forceps toward the work site before introducing the endoscope and also by assisting in macroscopic positioning; if a herniated disc is located caudal to the forceps, we flip the opening of our instrument toward that direction (i.e., at 9 o’clock of the patient’s anatomy clock, as shown in F). (E) The clock is drawn on the patient’s back; (G) directing the endoscope through the guidance of the clock drawn on. CR: cranial; LM: medial; CD: caudal.

The surgeon projects their hand on the video screen to guide themselves when starting the navigation (Figures 6A-6D).

If the procedure is performed on the patient’s right side, at 12 o’clock, there will be the spinal canal (posteromedial), at 6 o’clock the emerging root (anterolateral), at 3 o’clock the axilla of the root plus the inferior border of the superior pedicle (cranial), and finally at 9 o’clock, the upper border of the inferior pedicle (caudal). With those structures in view, the transversing nerve root should be seen running from 2 to 10 o’clock and the emerging root from 3 to 6 o’clock.

If, however, the procedure is performed on the patient’s left side, at 12 o’clock there will be the canal (posteromedial), at 6 o’clock the emerging root (anterolateral), at 3 o’clock the superior border of the inferior pedicle and at 9 o’clock the axilla of the root plus the inferior border of the inferior pedicle (cranial). As this view comes into place, the transversing nerve root will be seen running from 10 to 2 o’clock, and the emergent root from 9 to 6 o’clock.

Navigation

Navigational movement can be divided into three components: forceps positioning, triangulation, and joystick (hand movement).

Forceps Positioning

Prior to inserting the forceps into the endoscope, the surgeon should draw a watch on the patient’s back, allowing for calibration of the forceps. It is important to remember that the hand holding the forceps will serve as a compass. In the event that the surgeon becomes disoriented, they can locate themselves macroscopically by using whichever side the forceps are facing. Provided they keep the hand holding the forceps facing medially, even if the camera is rotated, they will know the disc is being grabbed medially regardless of how it appears on the video feed. Before entering, the forceps should be positioned in the previously drawn clock, then the surgeon will turn their hand caudally, introducing the forceps in that direction. With the alignment of the endoscope preserved, the forceps will open to the 9 o’clock mark on the screen. If the surgeon’s hand is kept facing the caudal region, he will know in which direction the endoscope is moving, regardless of the orientation the video feed shows.

Triangulation

Triangulation consists of creating an angle between the camera and the surgical instrument, allowing both to be used with the utmost safety by always visualizing the working area and adjacent structures. If the endoscope is aligned with the midline, and the forceps are being used toward the spinal canal, the surgeon won’t be able to see the working area. Therefore, by rotating the endoscope 90 degrees clockwise they will be able to see the distal region of the surgical instruments. It is worth noting that when the surgeon does this maneuver, the image shown on the screen turns 90 degrees counterclockwise. Strategically, by keeping their dominant hand positioned on the patient (in relation to the previously drawn clock), the surgeon will know the direction of the surgical field they are using. Thus, when working with the forceps facing cranially or caudally triangulation is created by the forceps (Figures 6E-6G).

When the forceps are facing medially or laterally, however, it is necessary to rotate the endoscope. In other words, when working in areas corresponding to the cranial or caudal aspect, triangulation, that is, the shift in viewing angle, is performed with the forceps. Rather, when working medially or laterally, this is performed by the endoscope.

Joystick (Hand Movement)

**Figure 7:**
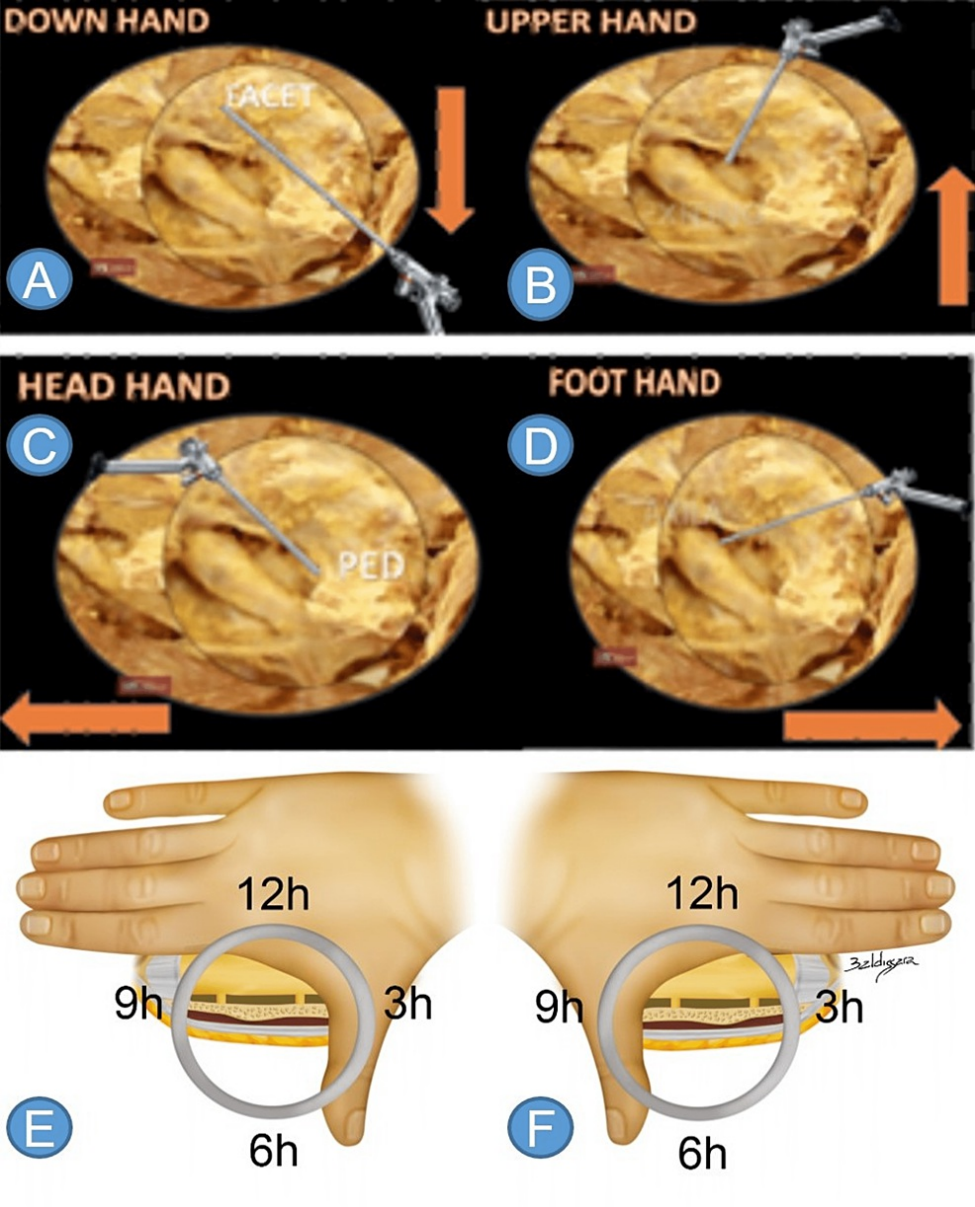
Joystick (hand movement), and cervical right and left-hand rule In endoscopic surgery, whenever we want to visualize one side, we must move the instrument in the opposite direction. The “down hand” movement (A) projects the endoscope to a posterior orientation, being used when we want to avoid the facet; the “upper hand” movement (B) is used when working laterally (emerging root); the “foot hand” movement (D) is used when dissecting cranially; the “head hand” movement (C) when dissecting caudally. “Down hand”: for central and posterolateral hernias. “Upper hand”: for foraminal and extraforaminal hernias. “Head hand”: for hernias in the pedicle. “Foot hand”: for hernias in the axilla. Hand projections for cervical spine surgery. The thumb represents the cervical root, and the other fingers represent the medulla. The cervical roots have a more perpendicular output in relation to the spine, placing the transversing nerve root from 11 to 7 o’clock on the left (F) and from 1 to 5 o’clock on the right (E). PED: pedicle. Source: author

“Joystick movement” is defined by the movement of the hand holding the endoscope to navigate through the foramen or vertebral canal. As an optics principle, whenever the surgeon needs to change to a specific direction, they must move their hand the opposite way of the desired direction. For example, if they wish to navigate cranially then they must move their hand caudally. If they need to work anterolaterally (extraforaminal region), they should move their hand posteromedially (Table 1, Figures 7A-7D).

**Table 1:** Composition of movements Forceps positioning, triangulation, and joystick (hand movement)

Localization	Forceps Positioning	Triangulation	Joystick (Hand Movement)
Axilla of the root	Cranial	Forceps	Caudal
Pedicle	Caudal	Forceps	Cranial
Central/ Ventral Facetectomy	Posterior	Endoscope	Anterior
Foraminal/ Extraforaminal	Anterolateral	Endoscope	Posteromedial

With the interlaminar approach, the same principles apply. To the patient’s right side, the surgeon extends their right hand forward, and the transversing nerve root is seen running from 4 to 7 o’clock, while the dural sac is observed from 2 to 10 o’clock. On the left side, the surgeon should extend their left hand, bringing the transversing nerve root into view running from 7 to 4 o’clock and the dural sac from 2 to 10 o’clock.

In the cervical spine, the root follows a less oblique path. On the patient's right side, the surgeon projects their right hand, allowing the transversing nerve root to be identified running from 1 to 5 o’clock and the spinal canal from 2 to 10 o’clock. On the patient’s left side, the surgeon projects their left hand, thus bringing the transversing nerve root into view, running from 11 to 7 o’clock and the spinal cord from 10 to 2 o'clock (Figures 7E, 7F).

## Discussion

ESS has dramatically developed since the first attempts performed by Kambin et al. in 1970 [16]. Since the advent of modern ESS techniques, practice with cadaver models, among others, has been warranted [17]. Alas, mastery of endoscopic techniques does not come easily and requires exposure not only to the endoscopic techniques themselves but to their concepts applied to a shifted perspective of anatomy that most physicians may not be entirely familiar with.

Understanding the anatomy through the endoscope is the main roadblock of endoscopic surgery’s learning curve for beginner spine surgeons. By decomposing the navigation movement into three components, the specialist developing their endoscopic techniques can understand how to manage the surgical equipment and improve their knowledge of this “new anatomy.”

In summary, learning endoscopic spine surgery can be challenging. However, improving our models and teaching methods can make these techniques more accessible to aspiring surgeons. Better teaching methods will make gaining experience with cadavers and live models more effective, receiving preceptorship and proctorship training, and eventually instructing others in these techniques more feasible. This, in turn, can lead to increased benefits for patients who may benefit from these MI procedures.

To our knowledge, there are no published mnemonics and navigational demonstrations for learners. The study conducted by Lewandrowski et al. [18] highlights the lack of agreement regarding the most effective training methodology. Given that this issue has been recognized as a key area for future research, we believe that our work could serve as an initial step toward standardizing endoscopic spine education.

## Conclusions

The authors present a novel teaching method for endoscopic spine surgery, consisting of a mnemonic called “rule of the hand.” When associated with proper endoscopic navigation, this and similar techniques may greatly assist in decreasing both the learning curve and radiation exposure used in procedures. Large-scale studies involving experienced surgeons and trainees are required to properly measure and quantify the impact of this method on surgical practice.
